# Multirooted supernumerary tooth in the anterior maxilla: A rare presentation

**DOI:** 10.1002/ccr3.6101

**Published:** 2022-07-18

**Authors:** Snehashish Ghosh, Safal Dhungel, Bijay Jaiswal, Prabhash Roy, A. Thirumal Raj, Shankargouda Patil

**Affiliations:** ^1^ Department of Oral Pathology College of Medical Sciences Bharatpur Nepal; ^2^ Department of Oral and Maxillofacial Surgery College of Medical Sciences Bharatpur Nepal; ^3^ College of Medical Sciences Bharatpur Nepal; ^4^ Department of Oral Pathology and Microbiology Sri Venkateshwara Denta College and Hospital Chennai India; ^5^ Department of Maxillofacial Surgery and Diagnostic Sciences, Division of Oral Pathology, College of Dentistry Jazan University Jazan Saudi Arabia; ^6^ Centre of Molecular Medicine and Diagnostics (COMManD) Saveetha Dental College & Hospitals, Saveetha Institute of Medical and Technical Sciences, Saveetha University Chennai India

**Keywords:** anterior, maxilla, multirooted, supernumerary

## Abstract

Multirooted supernumerary tooth is a rare finding, which predisposes to various malocclusion. The present case report depicts the presence of a multirooted supernumerary tooth in the anterior maxilla just adjacent to the midline in a 24‐year‐old patient.

## Graphical abstract

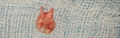

## 1 CASE PRESENTATION

**FIGURE 1:**
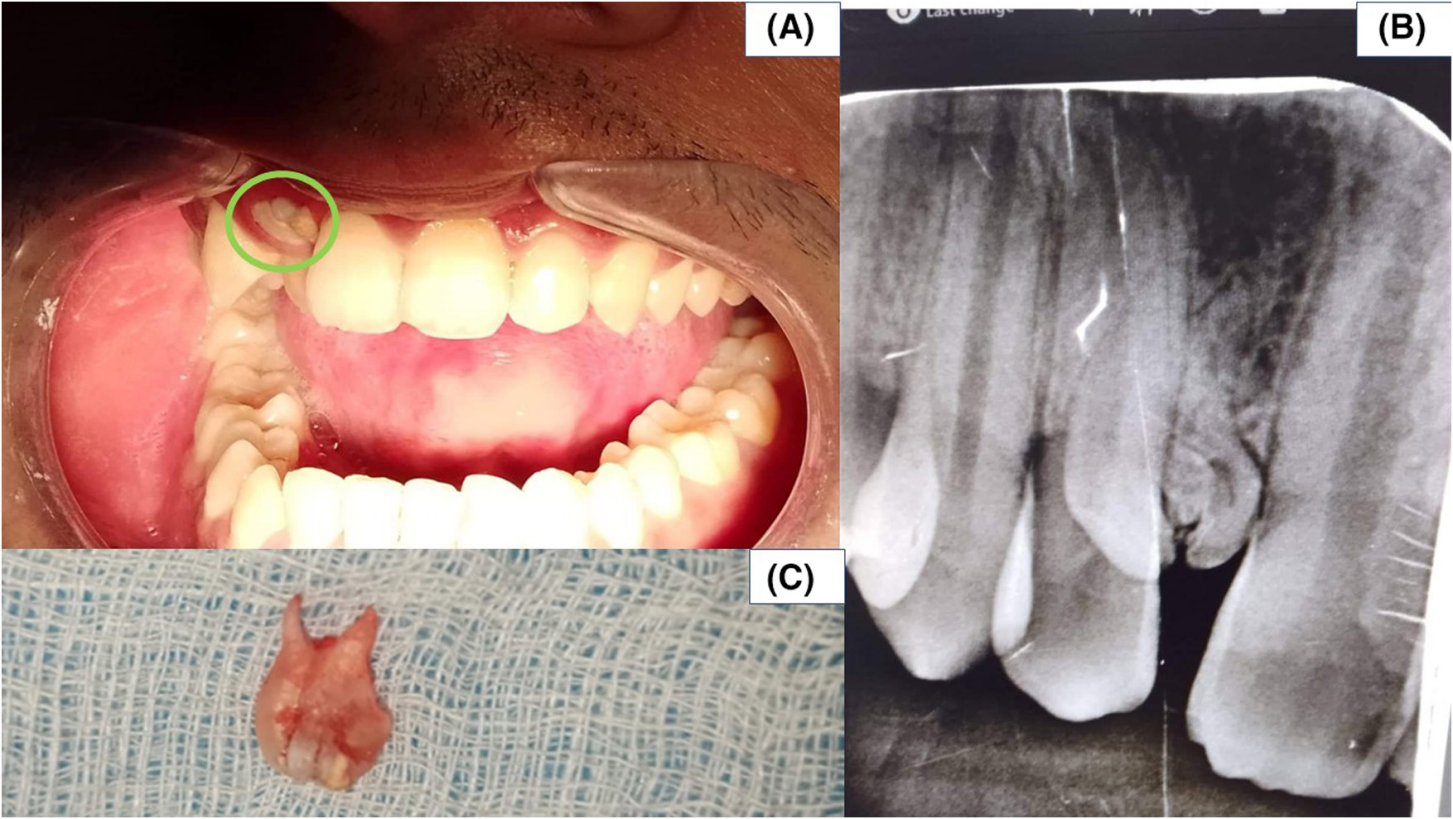
(A) Diffuse white mass, protruding from the maxillary gingiva, adjacent to the right maxillary central incisor. (B) Intra‐oral periapical radiograph showed a tooth‐like mass. (C) Excised mass showed a multirooted tooth partially resembling a primary molar

A 24‐year‐old male patient visited the outpatient department of the College of Medical Sciences, Bharatpur, Nepal, with a chief complaint of dirty teeth and wanted to get them cleaned. On examination, an irregular, diffuse white mass was seen protruding from the maxillary gingiva, adjacent to the maxillary right central incisor (Figure 1A). The maxillary left lateral incisor palatally erupted, canine drifted mesially.

After addressing his chief complaint, the patient was advised for an intraoral radiograph to get a definitive diagnosis of the white mass. The radiograph showed a tooth‐like mass although not resembling any particular tooth (Figure 1B). Correlating clinically and radiographically diagnosis of compound odontoma was made.

The calcified mass was approached through an intra‐oral incision and removed in toto. There was no lining associated with the mass. The excised mass showed a multirooted tooth partially resembling a primary molar (Figure 1C).

Multirooted supernumerary teeth in anterior maxilla is a rare finding and can not only compromise function, and esthetics but can also predispose to an array of malocclusions, dental caries, cysts, and tumors leading to potential complications.^1^ It is vital to report such relatively rare cases as they could aid in an early diagnosis and formulation of a prompt multidisciplinary treatment strategy, which could prevent the patient from developing complications and improve the overall quality of the dentition.^2^

## AUTHOR CONTRIBUTIONS

All the authors contributed to the writing of the manuscript.

## CONFLICT OF INTEREST

None.

## ETHICAL APPROVAL

Ethics approval was not required from the institution, following our country's law, as this was a case report.

## CONSENT

Written informed consent was obtained from the patient to publish this case image following the journal's patient consent policy.

## Data Availability

The data that support the findings of this article are available from the corresponding author upon reasonable request.
